# Paradoxical Eczematous Reaction in a Patient With IL-17 Inhibitor-Treated Psoriasis Vulgaris

**DOI:** 10.7759/cureus.60051

**Published:** 2024-05-10

**Authors:** Brett Brazen, Jessica Colon, Landon Hobbs, Carlos Nousari

**Affiliations:** 1 Dermatology, Broward Health Medical Center, Fort Lauderdale, USA; 2 Osteopathic Medicine, Dr. Kiran C. Patel College of Osteopathic Medicine, Nova Southeastern University, Fort Lauderdale, USA

**Keywords:** paradoxical eczematous reaction, secukinumab, il-17 inhibitor therapy, dmard therapy, psoriasis vulgaris

## Abstract

Psoriasis is a chronic dermatologic condition that oftentimes requires extensive trial and error with various topical and systemic therapies until improvement is achieved. Interleukin-17 inhibitors (IL-17i), such as secukinumab, have been utilized in the treatment of psoriasis due to their mechanism of action. As with all medications, IL-17 inhibitors possess adverse effects, the most common being infection, nasopharyngitis, and injection site reaction. However, one rare adverse event, the paradoxical eczematous reaction, has been known to occur among patients on biologics including IL-17 inhibitors. Although it is a rare occurrence, our paper stresses the importance of educating patients about this potential side effect, the benefits and risks of starting a biologic, and obtaining informed consent from the patient. We present a case of a 14-year-old male with recalcitrant psoriasis vulgaris who developed a paradoxical eczematous reaction while undergoing treatment with secukinumab.

## Introduction

Psoriasis vulgaris is a chronic inflammatory skin condition that is due to the upregulation of cytokines, one of them being interleukin-17 (IL-17), characterized by red, itchy, scaly plaques [1-3]. Treatments that target IL-17 have been of interest to diminish the immune response and lead to improvement of psoriasis. Interleukin-17 inhibitors (IL-17i) such as secukinumab, ixekizumab, brodalumab, and bimekizumab are utilized to treat dermatologic autoimmune conditions. The main mechanism of action of secukinumab and ixekizumab is to inhibit IL-17A directly, whereas brodalumab inhibits the IL-17A receptor and bimekizumab inhibits IL-17A and IL-17F [1,2]. Secukinumab, ixekizumab, brodalumab, and bimekizumab are approved for the treatment of psoriasis, psoriatic arthritis, and ankylosing spondylitis [1,2]. Amid the growing prominence of biologic therapies, it is crucial to acknowledge potential clinical reactions associated with their use. Various clinical trials have revealed common adverse events associated with these biologics. A meta-analysis involving 57 studies with a total of 28,424 patients with psoriasis treated with anti-IL-17 revealed that common adverse events were infection, nasopharyngitis, and injection site reactions [4]. Eczema was a rare adverse event that affected 67 out of 1,230 patients [4]. One such reaction, the paradoxical eczematous response triggered by IL-17 inhibitor biologics, has been well documented with various cases published [3,5,6]. We present a case of a 14-year-old male with recalcitrant psoriasis vulgaris who developed a paradoxical eczematous reaction while undergoing treatment with secukinumab.

## Case presentation

**Figure 1:**
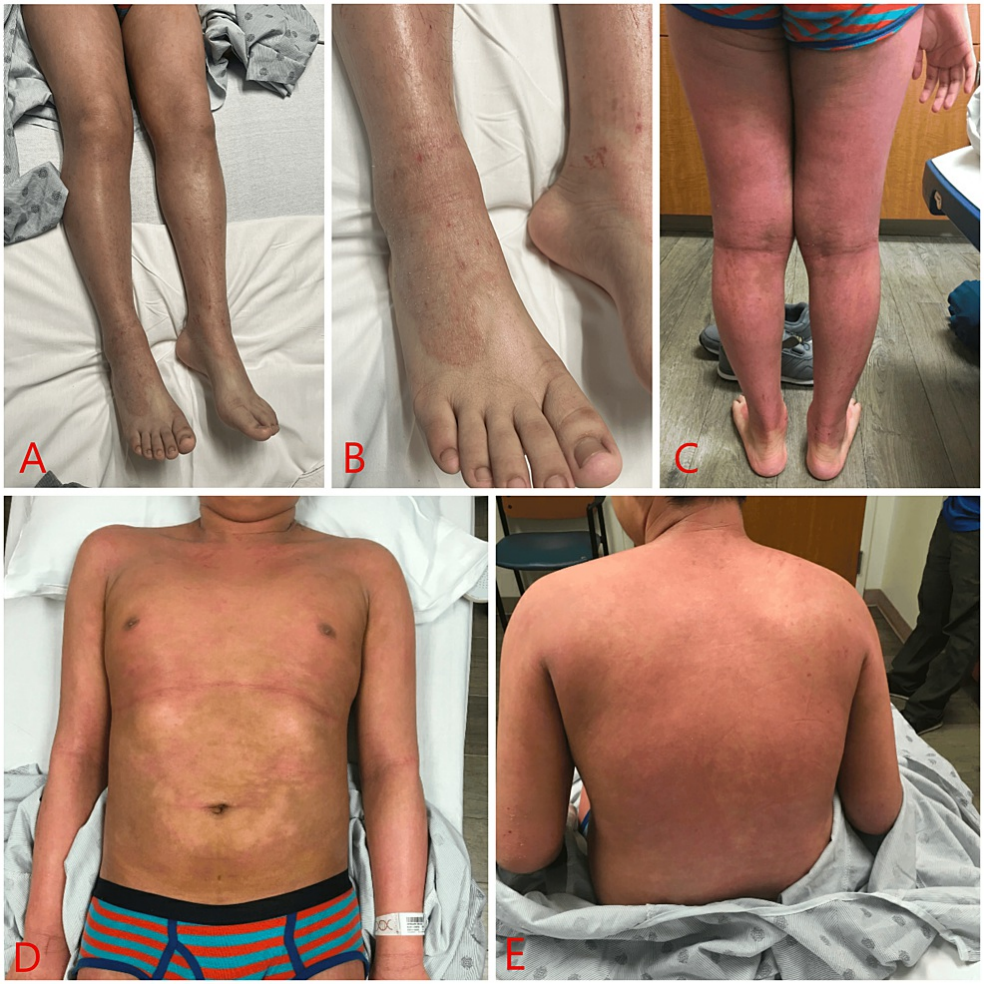
Diffuse, well-demarcated, erythematous rash with fine white scale and excoriations involving the anterior and posterior lower extremities (A-C) and anterior and posterior torso (D and E).

A 14-year-old male with a history of recalcitrant psoriasis vulgaris who was unresponsive to topical interventions with triamcinolone 0.1% ointment and wet wrap therapy was initiated on a three-month regimen of monthly 150 mg secukinumab injections. Following the first biologic dose, he developed a diffuse, scaly erythematous rash on the lower legs and abdomen, progressively extending to the upper extremities and posterior trunk, accompanied by intense pruritus that was intensified by subsequent treatments (Figure 1).

**Figure 2:**
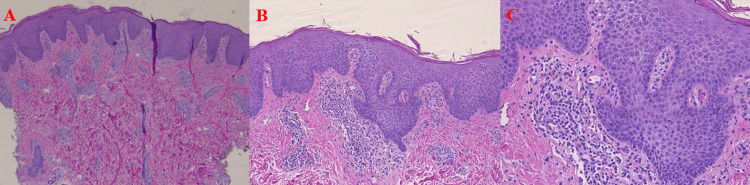
5×, 10×, and 20×: (A) Dense perivascular infiltrate with irregular acanthosis of the epidermis. (B) Compact eosinophilic stratum corneum with parakeratosis and irregular epidermal acanthosis. (C) Lymphocytic predominant perivascular infiltrate and ectatic superficial vessels in the papillary dermis with overlying irregular epidermal acanthosis.

**Figure 3:**
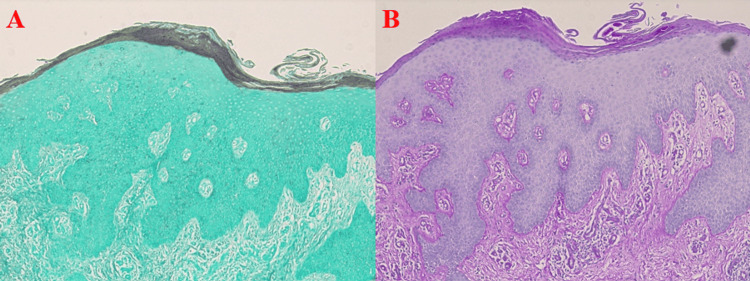
GMS and PAS: (A and B) Lack of fungal elements within the specimens, specifically (B) scant superficial vessels in the papillary dermis. GMS: Grocott/Gomori methenamine silver, PAS: periodic acid-Schiff

One month after his last injection, the patient presented to our facility with diffuse erythema and a fine exfoliative-like scale covering greater than 80% of his body surface area. Examination of the nails did not reveal psoriatic changes including oil spots, pitting, or onycholysis. There was no oral mucosal involvement, lymphadenopathy, or ocular findings. The patient was found to have moderately elevated eosinophilia of 20% and a significantly elevated IgE level of 49,970. Additionally, his antistreptolysin O (ASO) was positive with a titer of 400. Punch biopsies were performed for hematoxylin and eosin (H&E) and direct immunofluorescence (DIF), demonstrating confluent parakeratosis with decreased granular layer and irregular acanthosis without ectatic vessels or suprapapillary plate thinning. Superficial perivascular lymphocytic infiltrates with scattered eosinophils are present along with spongiosis, consistent with eczematous dermatitis. Immunofluorescence studies were negative (Figure 2 and Figure 3). Pulse-dose methylprednisolone 2 mg/kg divided into two doses for three days was initiated, and rapid improvement in both erythema and scaling was achieved. Nevertheless, diffuse xerosis remained, likely attributed to the prolonged half-life of the IL-17 inhibitor. Therefore, after completion of the pulse steroid, the patient was initiated on cyclosporine 75 mg daily for five days to ameliorate the persistent adverse reaction. Routine laboratory monitoring revealed no abnormalities in magnesium, lipids, blood urea nitrogen (BUN), creatinine, or uric acid.

## Discussion

While biologic therapies (IL-17 inhibitors, IL-23 inhibitors, and IL-12/IL-23 inhibitors) have become a mainstay in the treatment of psoriasis vulgaris, adverse effects are not uncommon. Psoriasis and atopic dermatitis are T-cell-mediated inflammatory diseases of the skin that have specific cytokines that trigger each condition. T helper 1 (Th1), Th17, and IL-17 are cytokines that have been identified as largely contributing to psoriasis, whereas Th2, IL-4, and IL-13 overproduction have been known to cause atopic dermatitis [7]. Researchers have suggested that when administering an IL-17 inhibitor, the Th1 and Th17 cytokines are diminished, which results in an imbalance in the Th2 immune response, causing a paradoxical eczematous reaction [5]. Due to dampening the cytokines that trigger psoriasis, there is a shift to cytokines favoring the opposing cutaneous condition of atopic dermatitis. Consequently, in an attempt to ameliorate the primary condition, biologics have the potential to trigger a new condition.

As a result, when choosing a biologic, especially in the pediatric population, it is important to discuss possible adverse reactions that may result from the treatment. Although paradoxical eczematous reaction is a rare side effect that results from biologics, there is still a risk of it occurring. Our patient developed a robust paradoxical eczematous reaction after undergoing IL-17 inhibitor therapy with secukinumab. Several cases have been published in which a similar eczematous reaction occurred after the administration of an IL-17 inhibitor for psoriasis vulgaris [3,4,6]. In these cases, the IL-17 inhibitor was discontinued, and patients were treated with prednisone and cyclosporine, which resulted in symptom improvement. It is paramount that patients are educated about this reaction and understand the benefits and risks of starting the medication and that informed consent is obtained. If a paradoxical eczematous reaction occurs, stopping the medication versus treating it through symptomatic control can be taken into consideration depending on patient severity, discussion with the patient, and informed consent of what the patient wishes to do. Some individuals may defer biologic discontinuation due to reaching progress with their condition after failing other therapeutic options. This case highlights the importance of recognizing such an adverse reaction when informing a patient about treatment options, particularly in light of the continuously evolving therapeutic research and the increasing use of biologics in clinical dermatology.

## Conclusions

Our case underscores the importance of understanding the underlying pathophysiology of paradoxical eczematous reactions. It also serves to further describe the diverse and rare spectrum of adverse effects linked to biological therapies. Most importantly, it emphasizes the importance of educating patients on these rare adverse effects and considering the benefits and risks of continuing or discontinuing therapy.
